# *Campylobacter portucalensis* sp. nov., a new species of *Campylobacter* isolated from the preputial mucosa of bulls

**DOI:** 10.1371/journal.pone.0227500

**Published:** 2020-01-10

**Authors:** Marta Filipa Silva, Gonçalo Pereira, Carla Carneiro, Andrew Hemphill, Luísa Mateus, Luís Lopes-da-Costa, Elisabete Silva

**Affiliations:** 1 CIISA - Centro de Investigação Interdisciplinar em Sanidade Animal, Faculdade de Medicina Veterinária, Universidade de Lisboa, Lisboa, Portugal; 2 Institute of Parasitology, Vetsuisse Faculty, University of Bern, Berne, Switzerland; Universidade de Coimbra, PORTUGAL

## Abstract

A new species of the *Campylobacter* genus is described, isolated from the preputial mucosa of bulls (*Bos taurus*). The five isolates obtained exhibit characteristics of *Campylobacter*, being Gram-negative non-motile straight rods, oxidase positive, catalase negative and microaerophilic. Phenotypic characteristics and nucleotide sequence analysis of 16S rRNA and *hsp60* genes demonstrated that these isolates belong to a novel species within the genus *Campylobacter*. Based on *hsp60* gene phylogenetic analysis, the most related species are *C*. *ureolyticus*, *C*. *blaseri* and *C*. *corcagiensis*. The whole genome sequence analysis of isolate FMV-PI01 revealed that the average nucleotide identity with other *Campylobacter* species was less than 75%, which is far below the cut-off for isolates of the same species. However, whole genome sequence analysis identified coding sequences highly homologous with other *Campylobacter* spp. These included several virulence factor coding genes related with host cell adhesion and invasion, transporters involved in resistance to antimicrobials, and a type IV secretion system (T4SS), containing virB2-virB11/virD4 genes, highly homologous to the *C*. *fetus* subsp. *venerealis*. The genomic G+C content of isolate FMV-PI01 was 28.3%, which is one of the lowest values reported for species of the genus *Campylobacter*. For this species the name *Campylobacter portucalensis* sp. nov. is proposed, with FMV-PI01 (= LMG 31504, = CCUG 73856) as the type strain.

## Introduction

The *Campylobacteraceae* family of the order *Campylobacterales* is the largest and most diverse family in class *Epsilonproteobacteria* of the phylum *Proteobacteria* [1]. *Campylobacter*, the type genus of the family, contains species known to be pathogenic to humans [2] and other animals [3] as well as non-pathogenic species that colonize a large range of molluscs, reptiles, birds and mammals [4]. Presently, the *Campylobacter* genus comprises 31 species and 13 subspecies [5]. Cells of most *Campylobacter* species are motile, microaerophilic, Gram-negative, slender, spirally curved rods and 0.5–5 μm long by 0.2–0.8 μm wide. However, some species exhibit straight rod morphology [1] and *C*. *gracilis*, *C*. *hominis*, *C*. *ureolyticus* and *C*. *blaseri* are non-motile [6–9]. Seven species colonize cattle [4], of which *C*. *coli*, *C*. *hyointestinalis*, *C*. *jejuni*, *C*. *lanienae* and *C*. *ureolyticus* are found in faeces [10–12], *C*. *sputorum* is a commensal of the penile and preputial mucosae [13] and *C*. *fetus* includes two subspecies with clinical relevance in cattle. The *C*. *fetus* subspecies *fetus* colonizes the bovine intestinal tract, causing sporadic abortion, whereas the subspecies *venerealis* inhabits exclusively the genital tract of cattle, and is the etiologic agent of Bovine Genital Campylobacteriosis [14]. Herein, we describe a new species of *Campylobacter* isolated from a beef herd with history of reproductive failure compatible with Bovine Genital Campylobacteriosis, in the Alentejo province of Portugal.

## Material and methods

### Ethics statement

The *in vivo* samples used in this study were obtained from a bull that performed natural mating in a herd with clinical signs of Bovine Genital Campylobacteriosis. The samples were collected by a certified veterinarian, using the recommended OIE sampling methods for diagnostic purposes. No ethical approval was required as this was part of a routine veterinarian evaluation of beef herd reproductive failure. *Ex vivo* samples were obtained from animals slaughtered for human consumption. As sampling was performed post-mortem, in a certified slaughterhouse, no ethical approval from an Institutional Animal Care and Use Committee (IACUC) or other relevant ethics board was required.

### Sampling, isolation procedures and culture conditions

Samples were obtained *in vivo* and *post-mortem*. The *in vivo* sample was collected from one mature Charolais bull, for laboratory diagnostic purposes. This sample was obtained from the preputial fornix using a technique combining scraping and small volume fluid washing (phosphate buffered saline; PBS) of the mucosa [15], and transported to the laboratory within 4 hours in two aliquots, one in Weybridge transport enrichment medium (TEM) and one in PBS. The bull had a normal routine breeding soundness evaluation and performed natural mating in a beef herd in the Alentejo province of Portugal. This herd showed fertility features compatible with Bovine Genital Campylobacteriosis, namely a low breeding season fertility, and late and spread calvings within the calving season. *Post-mortem* samples were also collected from the preputial fornix at two slaughterhouses and transported to the laboratory in PBS within 4 hours. The *in vivo* collected sample transported in Weybridge TEM was incubated at 37°C in a microaerobic atmosphere (Genbox Microaer, Biomérieux, France) for 48 hours, as an enrichment step. Enriched samples were plated through two different approaches: i) passively filtered onto blood agar (BA) and ii) spread in *Campylobacter* Skirrow Agar (CSA) [16]. In the BA approach, 0.65-μm mixed cellulose ester membrane filters (Advantec, Japan) were applied to the surface of BA plates supplemented with 5% sheep blood (Columbia agar + 5% sheep blood, Biomérieux) and inoculated with 100 μl of enriched sample for 30 minutes in aerobic conditions at room temperature; filters were then removed. In the CSA approach, 100 μl of enriched sample was spread on CSA plates. The BA and CSA plates were then incubated in microaerobic conditions at 37°C for 72 hours. The samples transported in PBS (*in vivo* and *post-mortem* collected) were diluted (ten-fold dilutions; 10^−1^, 10^−2^ and 10^−3^), streaked onto BA plates and incubated in a microaerobic atmosphere at 37°C for 48 hours. Colonies with *Campylobacter*-like morphology (small, smooth, translucent) were streaked onto BA and returned to a microaerobic atmosphere for a further incubation at 37°C for 48 hours. Before phenotypic and genotypic characterization, cells were microscopically examined using Gram staining.

### Molecular identification and phylogenetic analysis

Genomic DNA from bacterial isolates was extracted using DNeasy Blood and Tissue kit (Qiagen, Germany), according to the manufacturer’s instructions. The 16S rRNA gene was amplified and sequenced using a universal set of primers–fD1 and rP1 (Table 1). Additionally, the flanking regions were amplified and sequenced with the primer sets FrAF/Vc1-2 and FrBF/FrBR (Table 1). Primers (FrAF, FrBF and FrBR) were designed using Primer-BLAST [17], based on the whole genome sequencing data. Primers FrAF and FrBR were designed to target a neighbour sequence of the 16S rRNA gene in order to obtain a full-length sequence. PCR reactions were carried out in a 50 μl mixture containing 0.3 μM of each primer, 200 μM of deoxynucleotide-triphosphates (4you4 dNTP Mix, Bioron, Germany), 1x reaction buffer (Complete NH4 reaction Buffer, 10x, Bioron), 2 units of DFS-Taq Polymerase (Bioron) and 3 μl of DNA. The thermal cycle conditions were as follows: 94°C for 2 min, followed by 30 cycles of denaturation (94°C for 30 s), annealing (30 s), and extension (72°C for 60 s), with a terminal extension step of 72°C for 5 min. The annealing temperature set for each primer pair is shown in Table 1. The amplified sequences were aligned and trimmed to create a full-length 16S rRNA gene sequence. The nucleotide sequence of FMV-PI01 isolate (GenBank accession no: MN417497) was compared with other 16S rRNA gene sequences deposited in the NCBI database, using BLASTN algorithm. To investigate the taxonomic position of the bacterial isolates, a phylogenetic tree based on 1513 nucleotide positions of 16S rRNA gene sequences was reconstructed. Available sequences of 16S rRNA gene of other *Campylobacter* species were retrieved from the GenBank database for phylogenetic analysis with Molecular Evolutionary Genetic Analysis (MEGA) X software [18]. Sequences were aligned with Clustal W algorithm [19] and positions with missing data were trimmed. The phylogenetic tree was reconstructed by the neighbour-joining method and stability of grouping was estimated by bootstrap analysis, set for 1000 replications. To further refine the phylogenetic analysis, a phylogenetic tree based on *hsp60* gene (also known as *cpn60* and *groEL)* sequences was also reconstructed, as described above. The amplification and sequencing of *hsp60* gene were carried out using two primer pairs (hsp60_AF/hsp60_AR and hsp60_BF/hsp60_BR, Table 1) to obtain a 1472 bp long sequence.

**Table 1:** Primer sequences used for 16S rRNA and *hsp60* genes amplification.

Gene	Designation	Primers (5′-3′)	Annealing temperature	Amplicon size (bp)	Reference
16S rRNA	fD1	AGAGTTTGATCCTGGCTCAG	52°C	1475	[20]
	rP1	ACGGTTACCTTGTTACGACTT			
	FrAF	CGATTGAGCCAAGGGCTTTA	52°C	461	This study
	Vc1-2R	ACTTAACCCAACATCTCACG			[21]
	FrBF	ACACGTGCTACAATGGCATA	53°C	451	This study
	FrBR	TCTCTGAAAACTAAACAAGGATGA			
*hsp60*	hsp60_AF	AACTTTATGGTGGCGTTAAAA	52°C	1118	
	hsp60_AR	AGTTTCTGTTGCAGCACCTA			
	hsp60_BF	AGCTTAATGTTGTTGAGGGA	51°C	1085	
	hsp60_BR	TTACATCATACCACCCATAC			

### Biochemical characterization and growth conditions

For biochemical characterization, bacterial cultures grown in a microaerobic atmosphere at 37°C for 48 hours were used. Oxidase activity was determined with oxidase test sticks (Liofilchem, Italy) and catalase activity was evaluated by observation of bubbling formation on a 3% peroxide hydrogen solution within 5 seconds. Urease and hydrogen sulfide (H_2_S) production were assessed on Christensen Urea Agar (Liofilchem) and Triple Sugar Iron (TSI) Agar (Liofilchem), respectively. Additionally, commercial tests were used to evaluate nitrate reduction, hippurate hydrolysis (Liofilchem) and indoxyl acetate hydrolysis (Indoxyl strips, Sigma-Aldrich), following the manufacturer’s instructions. The growth on BA supplemented with 1% glycine, 2% NaCl, 3.5% NaCl, 0.04% Tetrazolium chloride (TTC) and on MacConkey agar was determined according to standardized procedures, previously described [22,23]. The evaluation of growth on anaerobic and aerobic atmospheres at 37°C, and microaerobic growth at 25°C, 37°C or 42°C, after 48 to 96 hours was also performed. The bacterial motility was assessed by the hanging drop technique [24], using bacterial suspensions in PBS after 48 hours of growth on BA.

Reference strains *C*. *fetus* subsp. *fetus* NCTC 10842, *C*. *fetus* subsp. *venerealis* NCTC 10354, *C*. *coli* CNET 068, *C*. *jejuni* subsp. *jejuni* NCTC 11168, and isolates identified as *C*. *sputorum* bv. *sputorum*, and *Proteus sp*. were used as controls in the tests described above.

### Electron microscopy

Electron micrographs were taken from a pure culture of isolate FMV-PI01. Preparations for electron microscopy were performed as previously described [25–27], followed by post-fixation in 2% osmium tetroxide and stepwise dehydration (30/50/70/90/100%) in ethanol. For transmission electron microscopy, samples were embedded in EPON812. Ultrathin sections (80 nm) were placed onto 300-mesh formvar-carbon-coated nickel grids (Plano, Wetzlar, Germany) and stained with Uranyless and lead citrate [27]. Specimens were viewed on a CM12 transmission electron microscope operating at 60 kV.

For scanning electron microscopy, fixed and dehydrated samples were resuspended in two changes of hexamethyl-disilazane (Sigma), sputter coated with gold, and inspected on a JEOL 840 scanning electron microscope operating at 25 kV.

### Whole genome sequencing and comparative genomic analysis

The genomic DNA was extracted from a pure culture of the isolate FMV-PI01, grown on BA over 48 hours, using the DNeasy Blood and Tissue kit (Qiagen, Germany). After the genomic library preparation, the generated DNA fragments were sequenced using the HiSeq 4000 System (Illumina), with 150 bp paired-end reading sequences and assembled using CLC Genomics Workbench version 11.0.1 (CLC bio, Denmark), at Stabvida (Caparica, Portugal). The assembled genome was annotated with the Rapid Annotation Using Subsystem Technology (RAST) 2.0 pipeline [28,29].

The average nucleotide identity (ANI) was calculated with the webserver JspeciesWS [30] for the isolate FMV-PI01 and other *Campylobacter* species. The G+C content was determined based on the whole genome sequence of isolate FMV-PI01.

The identification of Clustered Regularly Interspaced Short Palindromic Repeats (CRISPR) / CRISPR-associated (Cas) systems was performed using the CRISPRCasFinder webserver [31]. Additionally, the presence of putative virulence factor coding genes was evaluated based on the homology of translated sequences using BLASTP. Only sequences with query coverage >95% and identity >50% with known protein sequences of virulence factors were considered.

To estimate the pathogenic potential of this novel species, the assembled genome of isolate FMV-PI01 was analysed with the PathogenFinder Web server [32], using the automatic model option.

## Results and discussion

### Morphological characterization

The aliquot of the *in vivo* collected preputial sample transported in Weybridge TEM produced no *Campylobacter*-like morphology colonies on either approach (BA and CSA). In contrast, the *in vivo* collected preputial sample aliquot transported in PBS and streaked onto BA produced *Campylobacter*-like morphology colonies (isolate FMV-PI01). *Post-mortem* preputial samples, collected in PBS, produced four isolates (isolates FMV-PI02 to FMV-PI05) from beef bulls arising from four geographically distinct herds.

**Fig 1:**
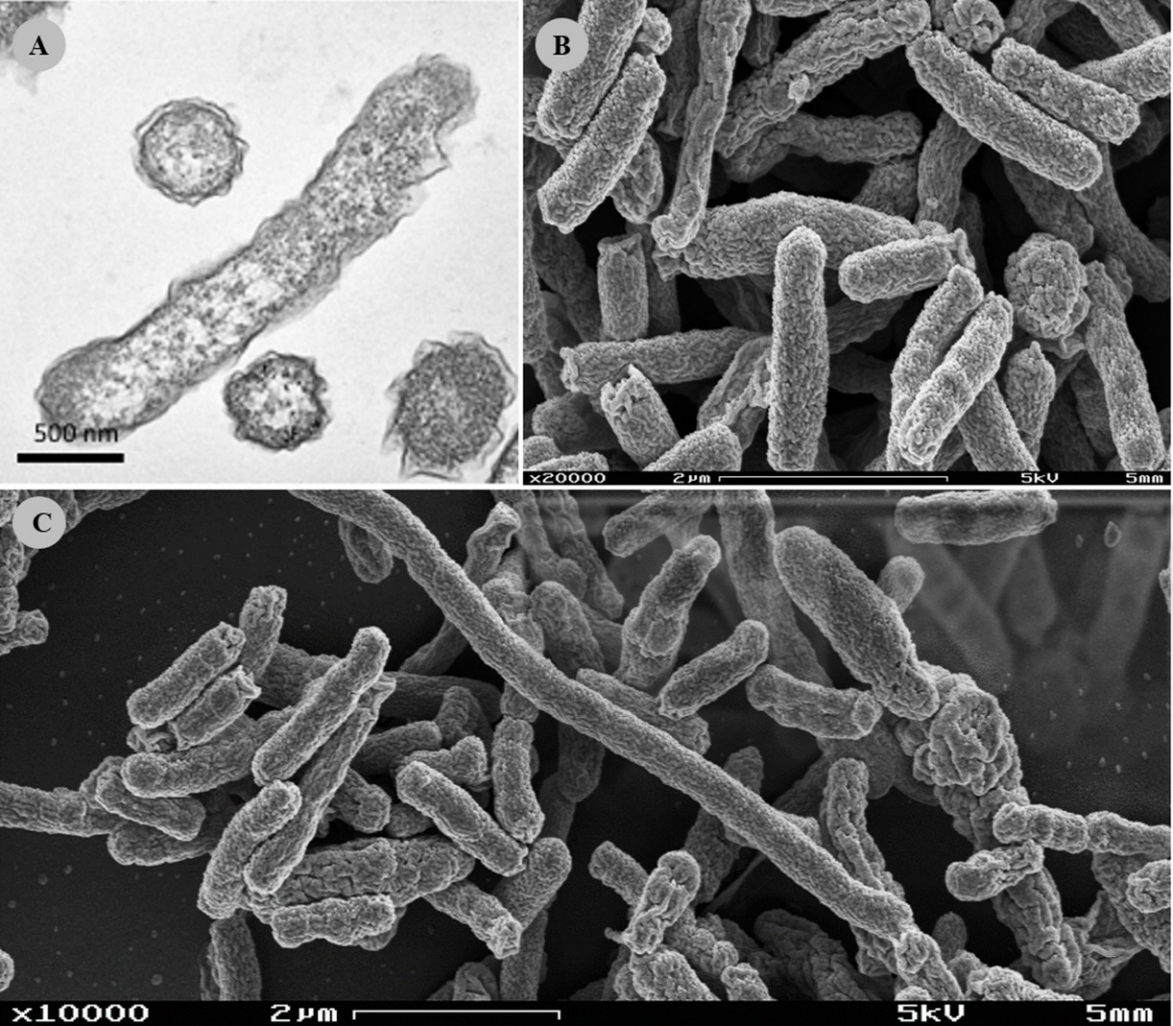
Electron micrographs of isolate FMV-PI01. (A) Transmission electron microscope micrograph in longitudinal and transverse views. Scale bar, 500 nm. (B) Scanning electron microscope micrograph in detail. Scale bar, 2 μm (C) Scanning electron microscope micrograph with evident long filamentous cells (white arrow). Scale bar, 2 μm.

Colony morphology in BA was common to all isolates, colonies being punctiform, circular, with convex elevation, smooth margins, without pigmentation and no haemolysis observable. Bacterial cells from all isolates were Gram-negative and exhibited a straight rod shape. Electron microscopy revealed that isolate FMV-PI01 bacterial cells had an average length of 1.68±0.07 μm (n = 30) and an average width of 0.44±0.01 μm (n = 30), and were devoid of a flagellum. Occasional filamentous cells were observed displaying lengths up to 18 μm (Fig 1).

### Phenotypic characterization

The phenotypic tests were validated by control *Campylobacter* species strains, whose results were all in agreement with those reported in the literature [33]. All five isolates presented identical phenotypic characteristics (Table 2). Isolates were positive for oxidase activity, a feature present in all *Campylobacter* species except *C*. *gracilis* and sporadic isolates of *C*. *concisus* and *C*. *showae* [1]. Isolates were negative for catalase activity and were unable to hydrolyse urea, hippurate and indoxyl acetate. From the seven *Campylobacter* species that colonize cattle, only *C*. *jejuni* is able to hydrolase hippurate [4,8]. Contrary to the majority of *Campylobacter* species, the isolates were unable to reduce nitrate. In fact, only three other species of the *Campylobacter* genus fail to reduce nitrate: *C*. *hominis*, *C*. *mucosalis* and *C*. *concisus* [1]. Production of H_2_S was not observed in TSI agar. The growth tests showed that the isolates were microaerophilic, although they could tolerate anaerobic conditions with weaker growth. The microbial growth in microaerobic conditions was similar to the majority of the *Campylobacter* species [1], observable at 37°C and 42°C, but not at 25°C. Further testing showed that four of the five isolates grew in the presence of 1% glycine, but none grew on BA supplemented with 2% or 3.5% NaCl. Growth was not observed either on MacConkey agar or on Muller Hinton Agar supplemented with 5% sheep blood. Unlike other *Campylobacter* species (*e*.*g*. *C*. *hominis and C*. *ureolyticus*) [34], the isolates did not require hydrogen (H_2_) to grow since the employed gas-generating sachet Genbox does not release hydrogen. No motility was observed on the hanging drop method preparation, which can justify the absence of colonies with *Campylobacter*-like morphology in the BA approach since that technique was developed to isolate motile *Campylobacter* spp. that can cross the 0.65-μm mixed cellulose ester membrane filters. To summarize, the five isolates were Gram-negative rods, microaerophilic and oxidase positive, which are phenotypic traits common to the genus *Campylobacter* [13]. However, the isolates were distinguishable from the most related species *C*. *sputorum*, *C*. *corcagiensis*, *C*. *blaseri*, *C*. *ureolyticus* and *C*. *geochelonis* (see genomic characterization), since unlike these species, the isolates could not reduce nitrate (Table 2).

**Table 2:** Phenotypic characteristics differentiating FMV-PI isolates from the other *Campylobacter* species. Taxa: 1—FMV-PI isolates (n = 5); 2—*C*. *hominis*; 3—*C*. *gracilis*; 4—*C*. *sputorum*; 5—*C*. *ureolyticus*; 6—*C*. *corcagiensis*; 7—*C*. *mucosalis*; 8—*C*. *concisus*; 9—*C*. *pinnipediorum* subsp. *pinnipediorum*; 10—*C*. *curvus*; 11—*C*. *rectus*; 12—*C*. *showae*; 13—*C*. *coli*; 14—*C*. *hyointestinalis* subsp. *hyointestinalis*; 15—*C*. *jejuni* subsp. *jejuni*; 16—*C*. *lanienae*; 17- *C*. *fetus* subsp. *venerealis*;; 18—*C*. *hepaticus*; 19—*C*. *avium*; 20—*C*. *canadensis*; 21—*C*. *cuniculorum*; 22—*C*. *geochelonis*; 23—*C*. *helveticus*; 24—*C*. *insulaenigrae*; 25—*C*. *lari* subsp. *lari*; 26—*C*. *peloridis*; 27—*C*. *subantarcticus*; 28—*C*. *upsaliensis*; 29—*C*. *volucris*; 30—*C*. *blaseri*; 31—*C*. *iguaniorum*; 32—*C*. *ornithocola*. Data for reference taxa were obtained from previous species descriptions [7–9,33,35–47]. + 90–100%; (+) 75–89%; v 26–74%; (-) 11–25%; - 0–10%; nd–not determined; w—weakly positive.

	1	2	3	4	5	6	7	8	9	10	11	12	13	14	15	16	17	18	19	20	21	22	23	24	25	26	27	28	29	30	31	32
Motility	-	-	-	+	-	+	+	+	nd	+	+	+	+	+	+	+	+	+	+	+	+	+	+	+	+	nd	nd	+	nd	-	nd	nd
Oxidase	+	+	-	+	+	+	+	v	+	+	+	v	+	+	+	+	+	+	+	+	+	+	+	+	+	+	+	+	+	+	+	+
Catalase	-	-	(-)	v*	v	+	-	-	+	-	(-)	+	+	+	+	+	(+)	+	w	v	+	+	-	+	+	+	+	-	+	+	+	-
α-haemolysis	-	-	-	+	v	-	-	(-)	+	(-)	+	+	(-)	v	+	+	V	-	-	-	+	-	+	nd	+	nd	+	+	nd	-	+	-
Urease	-	-	-	v*	+	+	-	-	+	-	-	-	-	-	-	-	-	-	-	v	-	-	-	-	-	nd	nd	-	nd	+	-	+
Hippurate hydrolysis	-	-	-	-	-	-	-	-	-	(-)	-	-	-	-	+	-	-	(+)	+	-	-	+	-	-	-	-	-	-	-	-	-	-
Indoxyl acetate hydrolysis	-	-	(+)	-	v	v	-	-	-	v	+	v	+	-	+	-	-	+	+	-	+	-	+	-	-	nd	-	+	-	+	-	-
Nitrate reduction	-	-	(+)	(+)	+	(+)	(-)	(-)	+	+	+	+	+	+	+	+	(+)	v	+	v	+	+	+	+	+	nd	nd	+	+	+	+	+
H_2_S production	-	-	-	+	-	+	+	-	+	(-)	-	v	-	+	-	-	-	-	-	v	-	-	-	+	-	nd	-	-	-	+	+	+
Growth in/at/on:																																
1% glycine	v	+	+	+	+	+	v	(-)	v	+	+	v	(+)	+	+	-	(-)	+	-	v	-	+	v	+	+	+	(+)	+	-	+	+	-
2% NaCl	-	nd	+	+	+	+	-	(-)	nd	v	v	+	-	-	-	nd	-	-	-	nd	-	+	-	-	+	(+)	+	-	-	nd	nd	nd
3.5% NaCl	-	nd	-	v	+	nd	-	-	nd	-	-	-	-	-	-	nd	-	-	nd	-	nd	nd	-	-	-	nd	-	-	nd	nd	nd	nd
MacConkey Agar	-	-	(+)	v	v	-	(+)	-	nd	(+)	-	+	v	v	-	+	V	-	-	+	-	-	-	nd	-	nd	(-)	-	w	+	nd	nd
TTC 0.04%	-	-	-	-	-	-	-	-	nd	V	-	-	+	-	+	nd	-	+	-	nd	v	-	-	nd	+	nd	nd	v	-	nd	nd	nd
25°C, microaerobic	-	-	-	-	-	nd	-	-	+	-	-	-	-	(-)	-	-	+	-	-	-	-	+	-	-	-	-	-	-	-	+	+	+
37°C, microaerobic	+	+	-	+	+	+	+	+	+	v	-	v	+	+	+	+	+	+	+	+	+	+	+	+	+	+	+	+	+	+	+	+
42°C, microaerobic	+	(-)	v	+	v	+	+	(+)	-	v	(-)	v	+	+	+	+	-	+	+	+	(+)	-	+	-	+	+	+	+	+	+	-	-
37°C, anaerobic	w	+	+	+	+	+	+	+	+	+	+	+	-	-	-	+	V	-	-	+	-	+	-	-	-	nd	+	-	+	+	+	+
37°C, aerobic	-	-	-	-	-	-	-	-	-	-	-	-	-	-	-	-	-	-	-	-	-	-	-	-	-	-	-	-	-	-	-	-
H_2_ requirement	-	+	+	-	+	-	+	+	-	+	+	+	-	v	-	-	-	-	v	-	-	-	-	nd	-	nd	-	-	nd	-	-	-

*test results differ between biovars.

### Phylogenetic analysis

The 16S rRNA gene sequence alignment revealed that the five bacterial isolates shared 99.87% sequence similarity. This homology, associated to their similar phenotypic characteristics, confirms that they belong to the same species. The comparative analysis of the 16S rRNA gene sequence of isolate FMV-PI01, using the BLASTN algorithm, confirmed that this isolate is closely related to the genus *Campylobacter*. The highest identities were obtained with *C*. *concisus* and *C*. *gracilis* (100% coverage and 94.7% identity) and *C*. *hominis* (100% coverage and 94.1% identity). These 16S rRNA sequence identities are below the threshold of 97%, defined for bacteria belonging to the same species [48], which supports the identification of a novel species within the *Campylobacter* genus.

**Fig 2:**
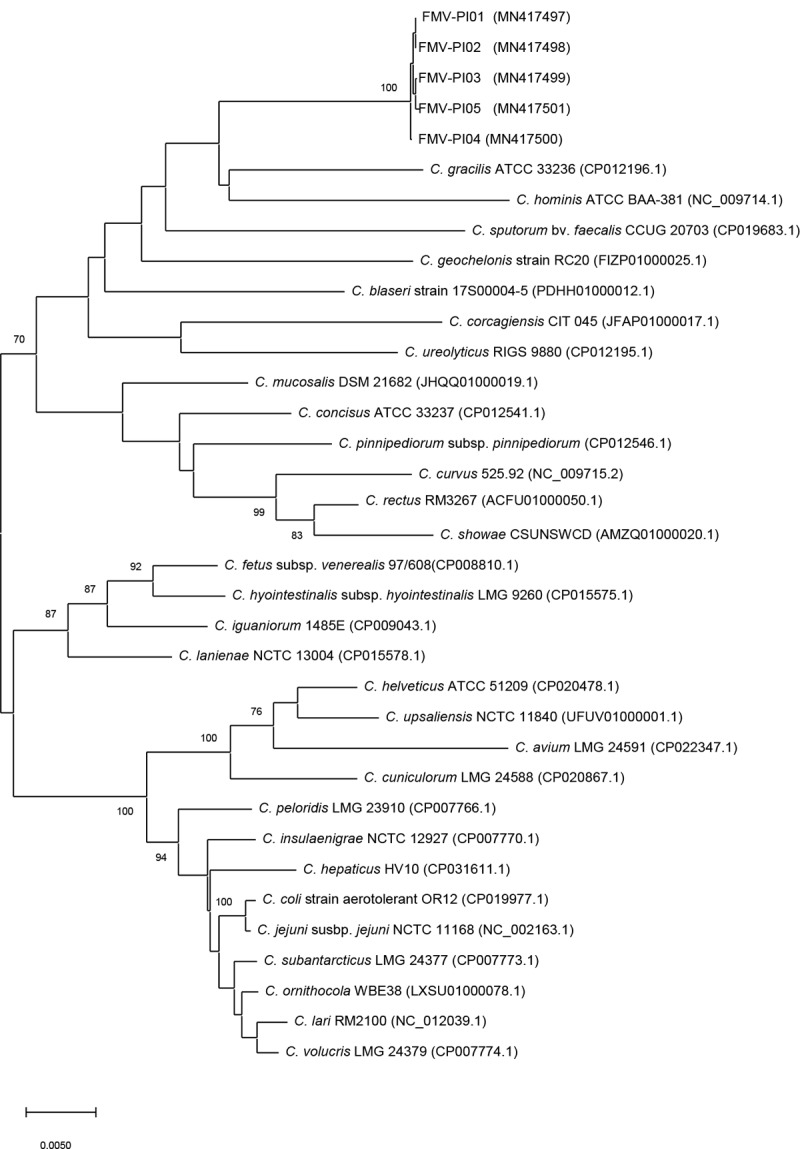
Phylogenetic tree based on 16S rRNA gene sequences of *Campylobacter* species, reconstructed by the neighbour-joining method. Bootstrap values (%) obtained from 1000 simulations are indicated at the nodes. Bootstrap values lower than 70% are not shown. Bar: 0.0050 substitutions per site.

**Fig 3:**
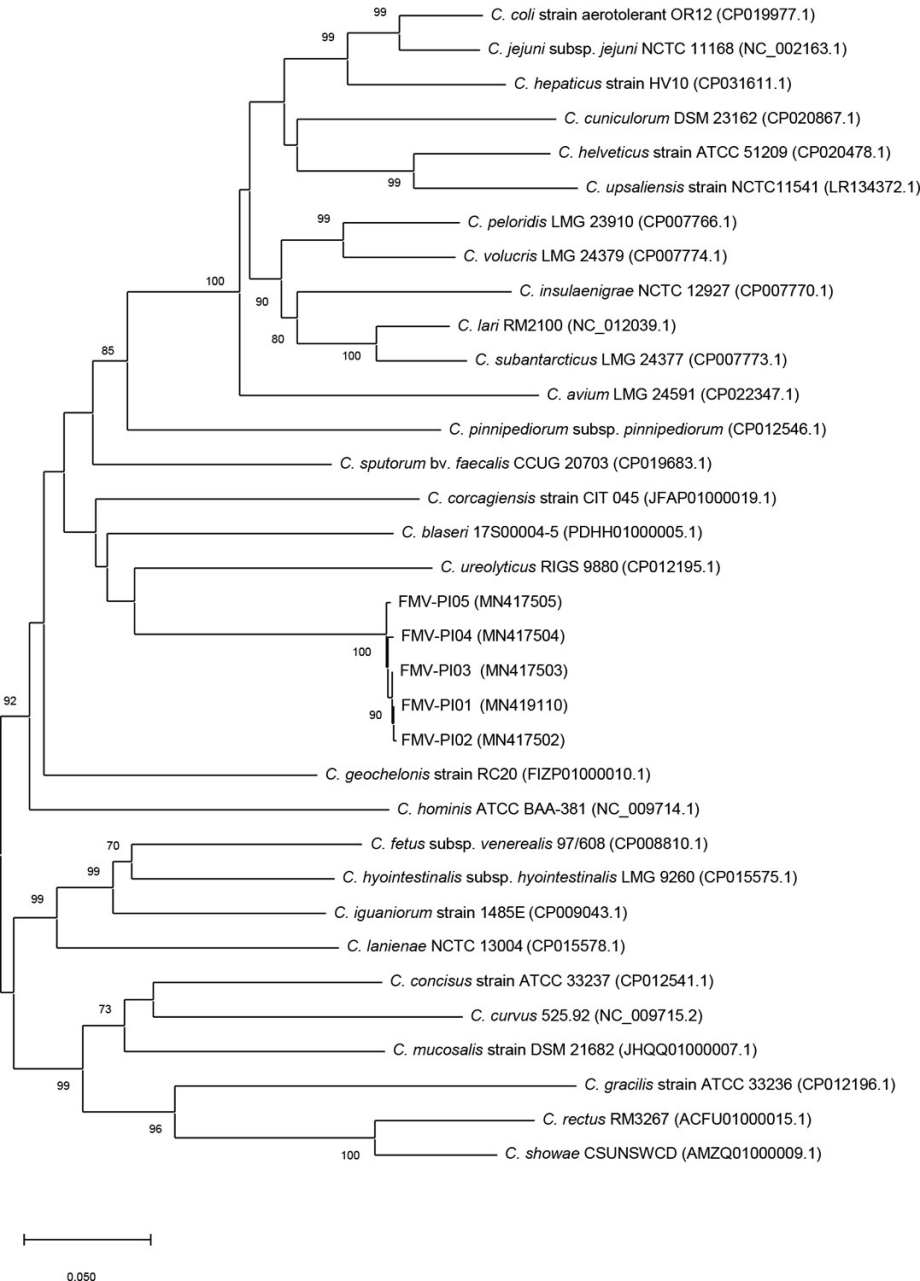
Phylogenetic tree based on *hsp60* gene sequences of *Campylobacter* species, reconstructed by the neighbour-joining method. Bootstrap values (%) obtained from 1000 simulations are indicated at the nodes. Bootstrap values lower than 70% are not shown. Bar: 0.050 substitutions per site.

The phylogenetic analysis using 16S rRNA and *hsp60* genes (Figs 2 and 3) demonstrated that these isolates form a robust cluster. However, the phylogenetic position of the five isolates was not clearly established based on the phylogenetic analysis. The low bootstrap values observed (< 70%) indicate that the 16S rRNA and *hsp60* genes have a weak discriminatory power relatively to the group of phylogenetically related species.

The 16S rRNA phylogenetic analysis identified *C*. *gracilis*, *C*. *hominis*, *C*. *sputorum*, *C*. *geochelonis*, *C*. *blaseri*, *C*. *corcagiensis* and *C*. *ureolyticus* as the most related taxa. This was also observed in the phylogenetic analysis based on *hsp60* gene, except for *C*. *gracilis*, which was not grouped with FMV-PI isolates. The *hsp60* gene analysis provided a better phylogenetic resolution, with a higher number of branches with high bootstrap values. Although the 16S rRNA gene is the most commonly accepted for use in taxonomic studies, phylogenetic analysis based on *hsp60* gene is more discriminative for some bacterial taxa as the family *Campylobacteraceae* [33,49,50]. A greater interspecies variation in the nucleotide sequence of *hsp60* gene than in 16S rRNA gene may explain these findings [50].

### Genomic characterization

The Whole Genome Shotgun project was deposited at DDBJ/ENA/GenBank under the accession VWSJ00000000. The version described in this paper is version VWSJ01000000. The genome of isolate FMV-PI01 is 1 767 933 bp long, composed of 98 contigs, with 28.3% G+C content. This G+C content is one of the lowest reported for species of the genus *Campylobacter*. The G+C content of a bacterial species may reflect adaptation to environmental niches and lifestyles, since non-free-living bacteria generally have shorter genomes with lower G+C content, promoting energy conservation in environments with scarcity of nutrients [51,52]. Interestingly, *C*. *sputorum*, also with one of the lowest G+C contents within the genus *Campylobacter* (29%) [53], colonizes the bull’s preputial mucosa, exhibiting niche preferences similar to herein described isolates.

The ANI analysis revealed that homology with other *Campylobacter* species was less than 75%, which is far below the 95–96% cut-off for isolates of the same species [54]. These nucleotide similarities are in accordance with the *hsp60* and 16S rRNA genes phylogenetic analysis results, showing that *C*. *ureolyticus*, *C*. *corcagiensis* and *C*. *blaseri* are the most closely related taxa with ANI values of 74.3%, 73.3% and 73.0%, respectively, followed by *C*. *geochelonis* (72.3%), *C*. *sputorum* (72.2%), *C*. *hominis* (70.1%) and *C*. *gracilis* (65.9%). The ANI values of these two latter species (*C*. *gracilis* and *C*. *hominis*) support the results of the analysis based on *hsp60* gene rather than the 16S rRNA gene. Overall, these findings support that the herein described isolates belong to the *Campylobacter* genus, representing a novel species, for which the designation *Campylobacter portucalensis* sp. nov. is proposed. The ANI analysis homology between *Campylobacter portucalensis* sp. nov. and its most related *Campylobacter* species is shown in Table 3.

**Table 3:** Average nucleotide identity (ANI) values (%) based on BLAST for *C*. *portucalensis* sp. nov. and the most related *Campylobacter* species. Strains: 1 –*C*. *portucalensis* sp. nov. FMV-PI01; 2 –*C*. *hominis* ATCC BAA-381; 3 –*C*. *fetus* subsp. *fetus* 82–40; 4 –*C*. *lari* RM2100; 5 –*C*. *insulaenigrae* NCTC 12927; 6 –*C*. *hyointestinalis* subsp. *hyointestinalis* LMG 9260; 7 –*C*. *hepaticus* HV10; 8 –*C*. *jejuni* subsp. *jejuni* LMG 9872; 9 –*C*. *corcagiensis* CIT 045; 10 –*C*. *blaseri* 17500004–5; 11 –*C*. *ureolyticus* DSM 20703; 12 –*C*. *sputorum bv*. *faecalis* CCUG 20703; 13 –*C*. *gracilis* ATCC 33236; 14 –*C*. *concisus* ATCC 33237; 15 –*C*. *geochelonis* RC20.

	1	2	3	4	5	6	7	8	9	10	11	12	13	14	15
**1**	*	70.1	69.8	69.7	69.6	69.6	69.3	69.3	73.3	73.0	74.7	72.2	65.9	68.4	72.3
**2**	70.2	*	69.2	68.3	68.2	69.1	68.2	68.3	69.8	69.7	71.6	70.2	68.2	68.3	70.2
**3**	69.4	68.5	*	68.2	67.9	78.5	67.6	67.8	68.8	69.4	69.3	69.5	66.6	68.2	69.7
**4**	70.0	68.5	68.9	*	81.9	68.8	74.3	74.5	69.0	69.8	69.8	69.8	65.5	68.0	69.0
**5**	69.7	68.3	68.4	81.9	*	68.4	74.1	74.0	68.6	69.5	69.6	69.3	65.4	67.5	68.5
**6**	69.1	68.4	78.6	67.9	67.7	*	67.4	67.8	68.7	69.0	69.2	69.5	66.7	68.6	69.7
**7**	69.4	68.2	68.5	74.5	74.5	68.2	*	84.4	68.6	69.1	69.3	69.2	65.1	67.0	68.1
**8**	69.1	68.1	68.1	74.4	73.9	68.3	84.4	*	68.5	68.8	69.1	68.9	65.6	67.6	68.4
**9**	72.3	68.9	68.6	67.8	67.8	68.5	67.5	67.7	*	71.2	73.0	70.3	65.4	67.7	71.1
**10**	72.5	69.3	69.2	68.9	68.6	69.2	68.3	68.3	71.8	*	72.8	71.2	65.4	67.9	72.6
**11**	74.8	71.6	69.8	69.7	69.4	69.8	69.1	69.2	73.9	73.1	*	72.0	65.8	68.6	72.3
**12**	71.9	70.2	69.8	69.4	69.1	69.8	68.8	68.8	71.2	71.5	71.8	*	66.2	68.8	70.9
**13**	65.5	67.2	66.2	64.6	64.4	66.4	64.4	64.8	65.2	64.7	64.8	65.4	*	67.7	67.0
**14**	67.7	67.4	68.3	67.0	66.6	68.6	66.5	66.9	67.7	67.8	67.81	68.2	68.0	*	69.0
**15**	71.4	69.2	69.3	67.9	67.2	69.3	67.1	67.5	71.0	72.1	71.33	70.2	67.3	68.8	*

**Fig 4:**
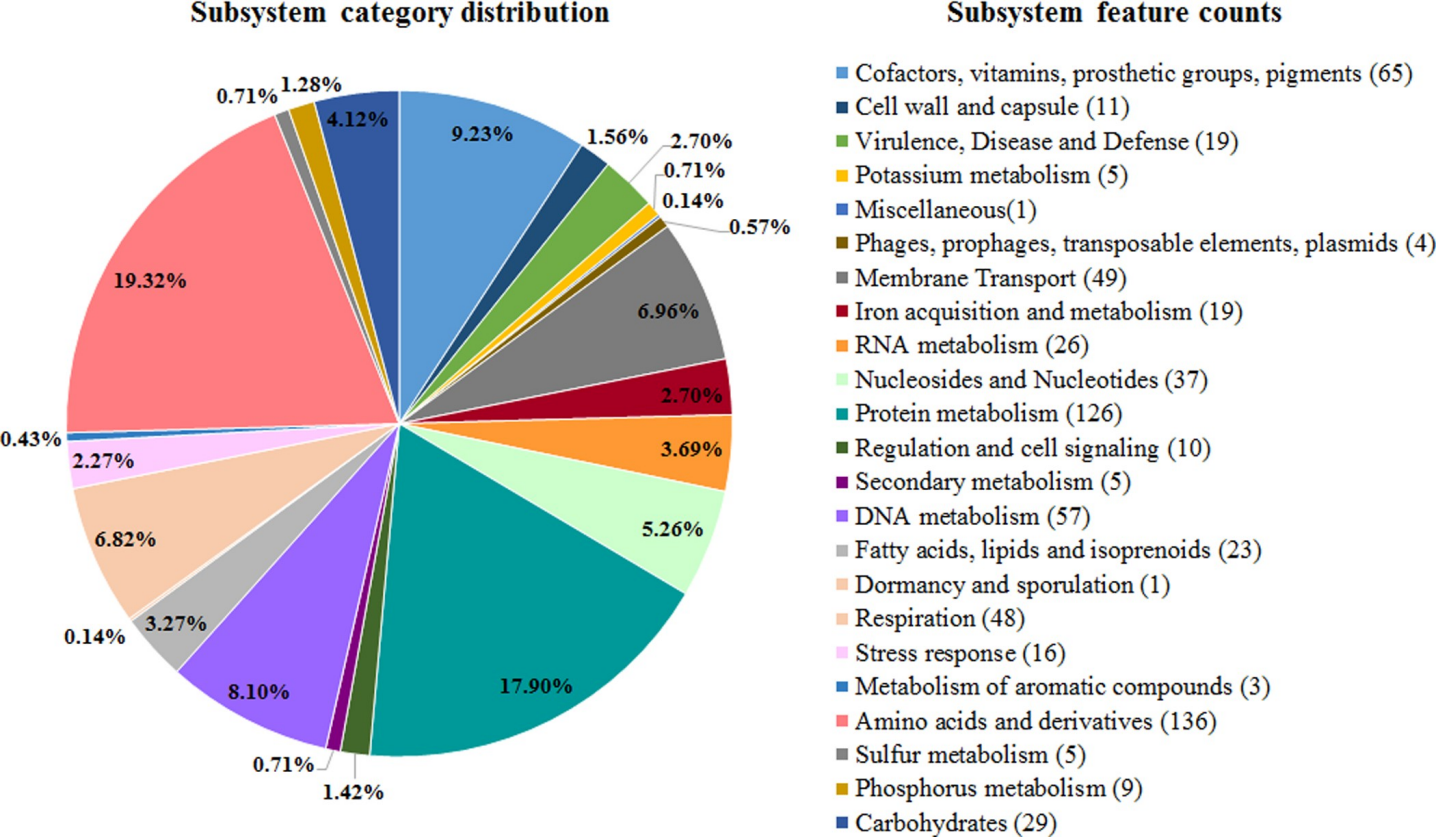
Subsystem category distribution in the genome of isolate FMV-PI01, based on the RAST server.

A total of 1877 coding sequences (CDS) and 41 RNAs (36 transfer RNA and 5 ribosomal RNA genes) were identified in FMV-PI01’s genome. Of the identified CDS, 497 were assigned to 191 subsystems. Subsystems with higher number of genes are related to metabolic processes, and include “protein metabolism” (n = 126), “amino acids and derivatives” (n = 136) and “cofactors, vitamins, prosthetic groups and pigments” (n = 65) (Fig 4). Nineteen genes involved in “Virulence, disease and defence” and 16 genes related to “stress response” were identified (Fig 4).

The CRISPRCasFinder identified one CRISPR-Cas system in FMV-PI01’s genome, which contains 15 CRISPR repeats with 13 CRISPR spacers and a type III-D Cas system. Despite CRISPR-Cas systems have been associated with phage defence mechanisms, these systems may also play a role in bacterial virulence and host immune evasion [55]. The diversity of CRISPR-Cas systems within the *Campylobacter* genus is wide, being the Cas systems type I and II the most common among *Campylobacter* species [55]. However, the type III-D, which was found in the genome of *C*. *portucalensis* sp. nov., was also identified in *C*. *fetus*. The mechanisms behind the selection of each system in different bacterial species [56], as well as the role of Cas system type III-D in *C*. *fetus*, remain unclear.

A more detailed analysis using BLASTP algorithm allowed the identification of genes potentially involved in adhesion and invasion to host cells. These genes encode homologous of the fibronectin/fibrinogen binding protein (98% query cover, 62% identity), collagenase like peptidase of U32 family (100% query cover, 81% identity) and *Campylobacter* invasion antigen B, ciaB (100% query cover, 64% identity). These virulence factor coding genes are present in several *Campylobacter* species, namely in *C*. *fetus* and *C*. *jejuni* [57]. Adhesion of *C*. *jejuni* to host cells is mediated by the fibronectin binding protein [58–60], and the mutational inactivation of the *ciaB* gene reduced the invasion in INT 407 cells, revealing that ciaB is involved in the internalization of *C*. *jejuni* [61,62]. Therefore, the presence of these genes in the genome of *C*. *portucalensis* is potentially related to host cell adhesion and invasion in the bovine reproductive tract. In addition, genes encoding multidrug efflux pumps of the resistance-nodulation-cell division family were found, namely a CmeABC efflux pump. These transporters are present in several species of *Campylobacter* (*e*.*g*. *C*. *jejuni*, *C*. *coli*, *C*. *fetus* and *C*. *lari*) and contribute to multidrug resistance [63]. For instance, this efflux pump in *C*. *jejuni* is involved in resistance to bile salts [64], macrolides, tetracycline [65], ciprofloxacin and other antimicrobials [66].

The *C*. *portucalensis* FMV-PI01’s genome encodes a type IV secretion system (T4SS), containing virB2-virB11/virD4 genes, highly homologous to the *C*. *fetus* subsp. *venerealis* T4SS (99% coverage and 97.2% identity). This T4SS was confirmed to contribute to *C*. *fetus* subsp. *venerealis* virulence properties, namely invasive and cytotoxic potential [67,68]. The fact that *C*. *portucalensis* sp. nov. and *C*. *fetus* subsp. *venerealis*, the causative agent of Bovine Genital Campylobacteriosis, share virulence factor coding genes and are both inhabitants of the bull preputial mucosa, may suggest that this novel *Campylobacter* species has the potential to cause disease in cattle. The herd from which the isolate FMV-PI01 was obtained presented signs of reproductive failure compatible with Bovine Genital Campylobacteriosis, the reason for the disease investigation. As in the above disease, where the bull acts as an asymptomatic carrier and signs of disease are only reflected on the female (embryonic and fetal mortalities) and herd (fertility rate, calving pattern and calving interval) sides [14], the bull from which the samples were taken was clinically sound. However, since several interacting factors may contribute to beef cattle herd’s infertility, one cannot conclude that the observed reproductive failure was the result of the infection with *C*. *portucalensis* sp. nov. To further investigate the pathogenic potential of *C*. *portucalensis* sp. nov in cattle fertility, research in the female reproductive tract needs to be addressed.

The evaluation of the pathogenic potential based on the PathogenFinder analysis showed that the probability of isolate FMV-PI01 being a human pathogen was 82.6%, indicating that this isolate may have the potential to cause disease in humans.

### Conclusion

The distinct phenotypic and genotypic characteristics of the bacterial isolates confirm the identification of a novel species within the *Campylobacter* genus, for which the name *Campylobacter portucalensis* sp. nov. is proposed. *C*. *portucalensis* sp. nov. is an inhabitant of bulls’ preputial mucosa with unknown pathogenic potential.

### Description of *Campylobacter portucalensis* sp. nov

*Campylobacter portucalensis* sp. nov. (por.tu.cal.en’sis. N.L. masc. adj. *portucalensis* referring to Portugal, from where the type strain was originally isolated).

In Columbia agar (supplemented with 5% sheep blood), after 48 hours in microaerobic atmosphere at 37°C, colonies are punctiform (1 mm in diameter), convex, circular with smooth margins and without any pigments. Colonies are non-haemolytic. Cells are Gram-negative, straight rods (length 1.68±0.07 μm and width 0.44±0.01 μm). A flagellum is absent and cells are non-motile. Occasional longer filamentous cells are observable.

Growth is observed on blood agar at 37°C under microaerobic and anaerobic (weak growth), but not aerobic conditions. Does not require H_2_ supplementation to grow. Isolates grow in microaerobic conditions at 37°C and 42°C but not at 25°C. Strains may differ in their ability to grow on blood agar medium supplemented with 1% glycine. Growth is not observed on blood agar medium supplemented with 2% and 3.5% NaCl. Unable to grow on Macconkey agar or Mueller-Hinton agar.

Phenotypically, *C*. *portucalensis* sp. nov. is oxidase positive and catalase negative. Negative for urease activity. Unable to hydrolyse hippurate and indoxyl acetate. Does not reduce nitrate. Does not produce hydrogen sulfide in TSI medium.

The genomic G+C content of the type strain is 28.3%.

The type strain FMV-PI01 (= LMG 31504, = CCUG 73856) was isolated from the reproductive tract of a bull (*Bos taurus*) sampled in the Alentejo province of Portugal in 2018.
